# Mitochondria transfer research from cellular mechanisms to immune–inflammatory and translational frontiers: a bibliometric analysis from 2006 to 2026

**DOI:** 10.3389/fimmu.2026.1898280

**Published:** 2026-08-19

**Authors:** Peiwei Su, Wanyu Luo, Chao Zhu, Xudong Zhufu, Xianyi Zhang, Haoxin Wu, Rui Xu, Haijun Zhao

**Affiliations:** 1 College of Traditional Chinese Medicine, Shandong University of Traditional Chinese Medicine, Jinan, China; 2 Nanjing University of Traditional Chinese Medicine, Nanjing, China

**Keywords:** immune regulation, inflammation, metabolic homeostasis, mitochondria transfer, mitochondria transplantation, mitochondrial quality control

## Abstract

**Background:**

Mitochondria transfer is an emerging mechanism of intercellular communication involved in mitochondrial homeostasis, metabolic remodeling, immune regulation, inflammatory responses, tumor progression, and mitochondrial transplantation-based therapy. However, the global research landscape and immune–inflammatory frontiers remain unclear.

**Methods:**

Publications on mitochondria transfer published between 1 January 2006 and 8 April 2026, were retrieved from the Web of Science Core Collection and Scopus. After deduplication and manual screening, 851 English-language articles and reviews were analyzed using bibliometrix, CiteSpace, VOSviewer, and Pajek.

**Results:**

The 851 publications included 566 articles and 285 reviews. They received 38,879 citations and an average of 45.69 citations per publication. Annual output increased markedly after 2018 and peaked in 2025. China and the USA were the leading contributors. Journal, co-citation, and keyword analyses showed that mitochondria transfer research has expanded from mitochondrial biology, stem cell–mediated repair, and cellular metabolism toward immune regulation, inflammation, tumor microenvironment remodeling, biomaterials, and translational medicine. Major knowledge bases and emerging hotspots included tunneling nanotubes, extracellular vesicles, mitochondrial transplantation, mitochondrial quality control, metabolic homeostasis, macrophages, T cells, B cells, immune evasion, macrophage polarization, and cGAS/STING-related mechanisms.

**Conclusion:**

Mitochondria transfer has developed into an interdisciplinary field connecting cellular mechanisms, immune–inflammatory regulation, disease microenvironment remodeling, and translational therapy. Future studies should clarify its molecular regulation and context-dependent consequences, particularly in immune cells, inflammatory diseases, tumor immune evasion, and mitochondrial transplantation-based interventions.

## 1 Introduction

Mitochondria are multifunctional organelles whose central function is to generate adenosine triphosphate (ATP) through oxidative phosphorylation, thereby providing energetic support for cellular metabolism and functional activity (1–3). Beyond energy production, mitochondria also coordinate diverse signaling pathways that are essential for maintaining cellular homeostasis (1). The coordination between mitochondrial structural integrity and dynamic functional processes provides an essential basis for maintaining mitochondrial functional stability and intracellular homeostasis (4). These processes include membrane potential homeostasis, ROS-dependent oxidative stress responses (5, 6), mitophagy, mitochondrial DNA (mtDNA) maintenance, mitochondrial protein synthesis, inter-organelle contacts, regulation of Ca²^+^ signaling, control of enzymatic activity, and metabolic signal transduction (1, 4). Within cells, mitochondria exist as dynamic networks and are precisely regulated by mitochondrial quality control systems, including mitochondrial biogenesis, fission and fusion, mitophagy, and proteostasis (4). For a long time, intracellular mitochondria were generally considered to originate primarily from vertical inheritance during cell division or to be renewed and expanded within cells through mitochondrial biogenesis (7). These classical regulatory mechanisms have been systematically described in previous studies (7, 8).

Over the past two decades, studies have shown that, in the absence of cell division, one cell can donate a subset of its mitochondria to another cell, a process known as horizontal or intercellular mitochondria transfer (7, 9). In 2006, Spees et al. first reported that mitochondria from adult stem cells or somatic cells could be actively and dynamically transferred to cells with mitochondrial dysfunction, thereby restoring impaired aerobic respiration (10). Subsequent studies reported that, in models of acute lung injury, bone marrow–derived stromal cells could transfer mitochondria to alveolar epithelial cells and thereby improve lung injury outcomes (11). Astrocytes were also shown to transfer functional mitochondria to neurons, contributing to neuroprotection after stroke (12). As research has progressed, the disease contexts and transport routes involved in mitochondria transfer have been increasingly elucidated. Mitochondria transfer can occur through diverse routes, including tunneling nanotubes (TNTs), extracellular vesicles, gap junctions, uptake of free extracellular mitochondria, and other mechanisms. It plays important roles in metabolic remodeling, immune regulation, inflammatory responses, tumor progression, and mitochondrial transplantation-based therapy (7, 13–17).

Although substantial mechanistic insights into mitochondria transfer have accumulated, and several narrative reviews have summarized its biological significance and application prospects from different perspectives, these studies are mainly qualitative and topic specific. A systematic, quantitative, and visual overview of the overall knowledge landscape of mitochondria transfer remains limited. In particular, the developmental trajectory, emerging hotspots, and immune–inflammatory research trends of this field have not been sufficiently mapped. Bibliometric methods use statistical approaches based on large-scale literature data to characterize relationships among published studies (18). This approach can reveal the developmental trajectory, knowledge base, and emerging trends of a research field across multiple dimensions, including annual publication output and citation frequency, collaboration networks at the country, institution, or author level, journal distribution, co-citation relationships, and keyword co-occurrence. Therefore, this study employed bibliometric and visualization analyses of publications on mitochondria transfer from 2006 to 2026. It systematically mapped, for the first time, the global research landscape of this field, including its developmental trajectory, collaboration networks, knowledge base, research hotspots, and future directions. By further integrating bibliometric evidence with immune–inflammatory mechanisms and translational implications, this study provides a systematic and data-driven complement to existing narrative reviews.

## 2 Methods

### 2.1 Search strategy, eligibility criteria, and data processing

The Web of Science Core Collection (WoSCC) is widely recognized as one of the most authoritative bibliographic databases for identifying high-quality scholarly publications (19). Scopus has likewise emerged as a major source of reliable scientific literature and research evaluation data (20). Indeed, WoSCC and Scopus have traditionally been the two most frequently used databases in bibliometric research (21). To enhance the robustness and comprehensiveness of the present bibliometric analysis, both WoSCC and Scopus were selected as literature sources, thereby enabling broader coverage of the relevant research landscape. PubMed was not included because it lacks a stable and directly usable cited references field for bibliometric analysis.

Following established bibliometric best practice procedures and with reference to the PRISMA framework, where applicable, the literature search and screening were conducted to ensure transparency and reproducibility (22, 23). Two independent researchers retrieved and screened records from WoSCC and Scopus on 8 April 2026, covering publications from 1 January 2006 to 8 April 2026. Because the literature search was conducted on 8 April, 2026, the 2026 data represent only a partial calendar year and are therefore presented as reference data when interpreting annual trends. The search strategy was developed based on the consensus recommendations for mitochondria transfer and transplantation nomenclature (9), and both databases were searched using title, abstract, and keyword fields. To ensure that the retrieval was focused on the mitochondria transfer field, the core search terms included “mitochondria transfer,” “mitochondrial transfer,” “intercellular mitochondria transfer,” “intercellular mitochondrial transfer,” “horizontal mitochondria transfer,” and “horizontal mitochondrial transfer.” Boolean operators were used to combine these terms, and database-specific search syntax was adapted for WoSCC and Scopus. To minimize retrieval noise, mitochondria transfer RNA-related terms were excluded, followed by manual review of the excluded records to avoid the loss of relevant studies. The detailed search strategies for both databases are provided in **Supplementary Data Sheet 1**. The inclusion criteria were as follows: (1) publications published between 1 January 2006 and 8 April 2026; (2) document types limited to articles and reviews; (3) English-language publications; and (4) studies focusing on mitochondria transfer or mitochondria transfer-related biological processes in biomedical, cellular, animal, or human research contexts. Records retrieved from WoSCC were exported in plain text format, whereas Scopus records were exported in CSV format. The open-source Python-based Bibliometric Data Consolidation Tool was then used to convert Scopus records into a WoS-compatible format, remove duplicates, and merge the datasets into a unified corpus suitable for subsequent bibliometric analysis.

**Figure 1:**
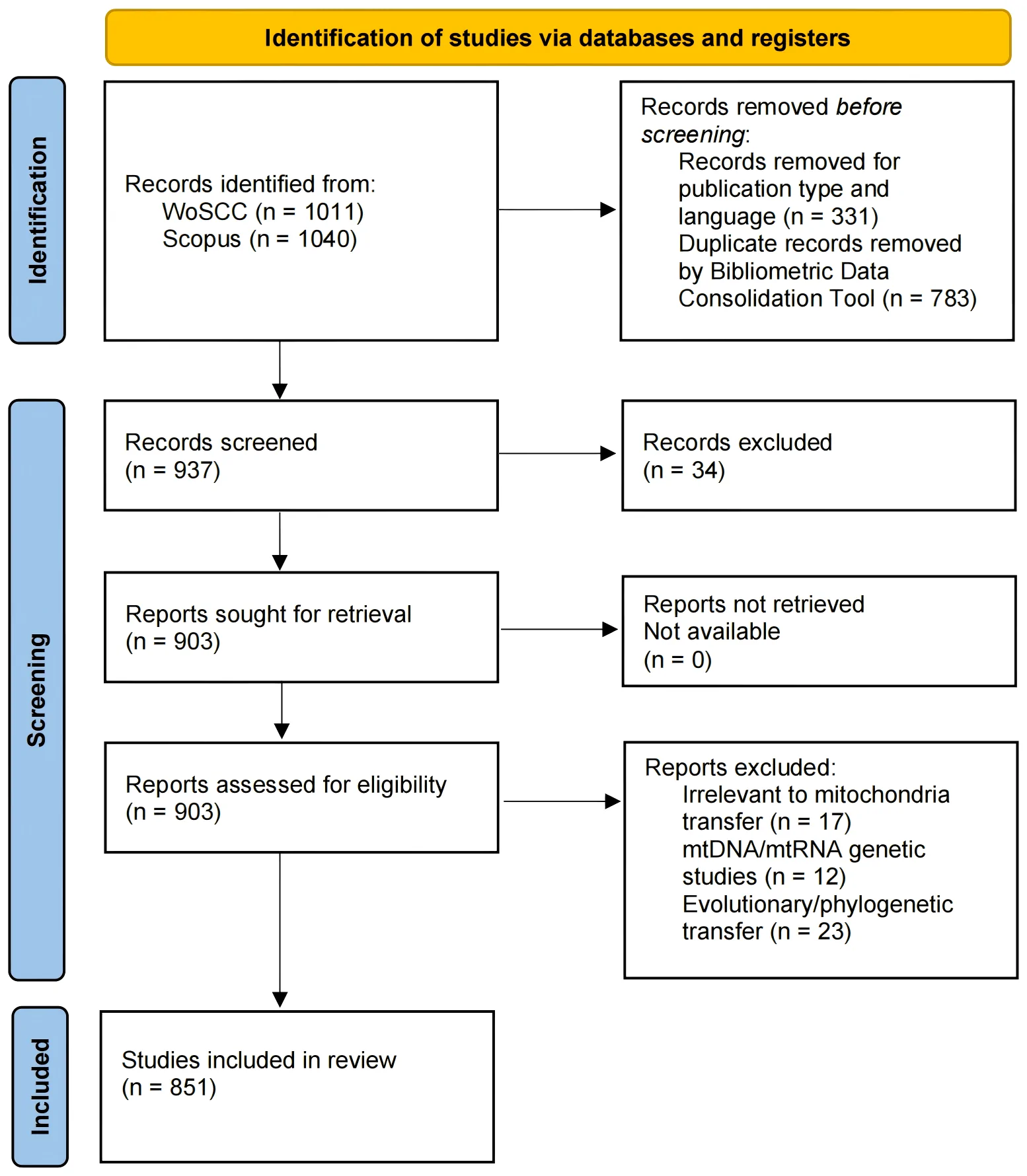
PRISMA-style flow diagram of literature search and screening for bibliometric analysis of mitochondria transfer research.

After format conversion, deduplication, and dataset merging, the combined records were subjected to independent manual screening. Records were excluded if they met any of the following criteria: (1) unrelated to mitochondria transfer based on the title, keywords, or abstract; (2) focused on mtDNA, mt-tRNA, mt-rRNA, or mitochondrial genetic variation without addressing mitochondria transfer as a biological process; or (3) referred to evolutionary, phylogenetic, hybridization-related, or horizontal genetic transfer rather than cell-biological transfer of intact mitochondria. Disagreements were resolved through consultation with scholars in the relevant field. The final verified dataset was used for bibliometric visualization and analysis. The full screening process, including database retrieval, duplicate removal, manual exclusion, exclusion reasons, and the final number of included publications, is summarized in the PRISMA-style flow diagram shown in **Figure 1**.

### 2.2 Data analysis and visualization

Bibliometric analysis and visualization in this study were performed using bibliometrix (version 5.3.0), CiteSpace (version 7.0, Advanced), and VOSviewer (version 1.6.20). The bibliometrix package is an open-source tool developed on the R platform for comprehensive science mapping and bibliometric analysis of scholarly literature (24). In the present study, it was primarily used for citation analysis and journal citation statistics.

CiteSpace is a Java-based bibliometric visualization tool for analyzing scientific literature within knowledge domains. It enables the construction of synthesized networks and their partition into distinct entity clusters, such as cited references, to facilitate the interpretation of knowledge structures and the identification of key research information (25). In this study, CiteSpace was used to analyze and visualize countries/regions, institutions, cited references, and keywords and to generate journal dual-map overlays.

VOSviewer is a bibliometric software tool for constructing and visualizing bibliometric networks based on datasets derived from databases such as WoS and Scopus (26). In the present study, VOSviewer was used to perform visual network analyses of authors, journals, and keywords. In addition, Pajek was used to further optimize the layout and visual presentation of clustered networks.

## 3 Results

### 3.1 Annual publication and citation trends in mitochondria transfer research

**Figure 2:**
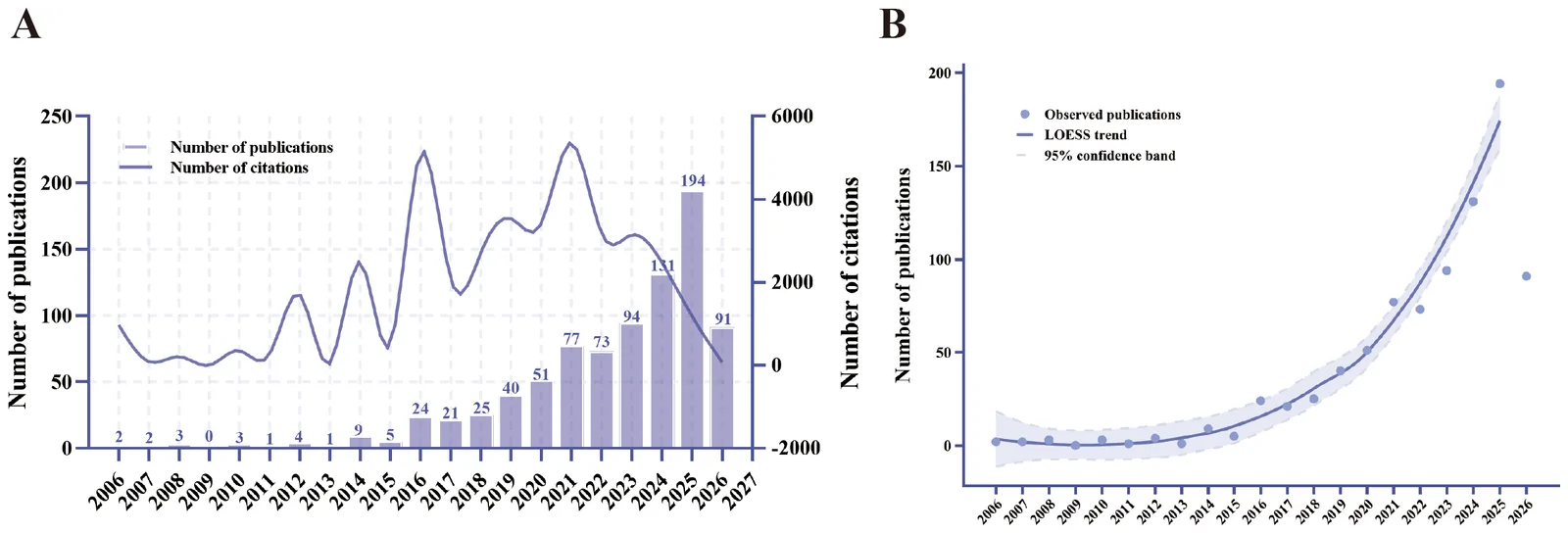
Trends in annual publications and citations of mitochondria transfer research from 2006 to 2026. **(A)** Annual publication and citation counts. **(B)** LOESS-fitted curve of annual publications with the 95% confidence band. The 2026 data include publications indexed up to 8 April 8 2026 and therefore represent partial-year data. These data are shown as reference data for the most recent stage of the field.

From 2006 to 2026, a total of 851 publications on mitochondria transfer were identified, including 566 articles and 285 reviews. These publications received 38,879 citations, with an average of 45.69 citations per publication (**Figure 2A**). Annual publication output remained low from 2006 to 2014, increased from 2015 onward, and accelerated markedly after 2018, reaching its highest level among complete calendar years in 2025. Because the search was conducted on 8 April 2026, the 2026 data represent a partial year and are presented for reference only. Annual citation counts also showed an overall upward trend, with notable peaks in 2016 and 2021, indicating increasing scholarly interest in this field. Consistently, the LOESS-fitted curve indicated a prolonged early period of slow growth followed by a sharp increase in publication output from around 2018, with particularly rapid growth after 2021 (**Figure 2B**). Overall, these findings suggest that mitochondria transfer research has attracted increasing scholarly attention and continues to expand in both scale and depth.

### 3.2 Country/region and institutional analysis of mitochondria transfer research

**Figure 3:**
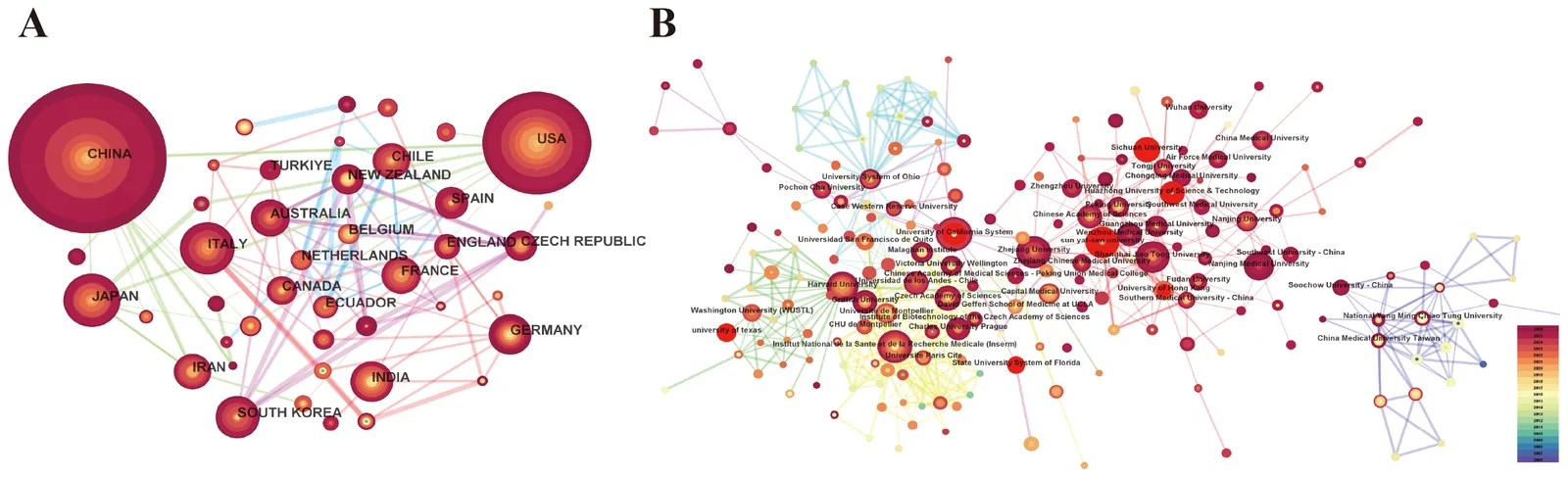
Collaboration networks of countries/regions and institutions in mitochondria transfer research. **(A)** Country/region-level collaboration network. **(B)** Institution-level collaboration network. Larger nodes indicate higher publication output, and links represent collaborative relationships. The colors indicate the temporal distribution of publications. The maps were generated using CiteSpace **(v)** 7.0.R0 with the following parameters: time span = 2006–2026, slice length = 1 year, node type = country/region for **(A)** and institution for **(B)**, selection criteria = g-index (*k* = 25), LRF = 2.5, L/N = 10, LBY = 5, e = 1.0, and pruning = none. Citation/frequency burst periods were highlighted in tree rings. No clustering analysis or additional minimum occurrence threshold was applied.

As shown in **Figure 3A** and **Table 1**, research on mitochondria transfer has involved broad participation across countries and regions, with China and the USA contributing the largest numbers of publications (373 and 198, respectively), followed by Japan (56), Italy (49), and France (37). In terms of betweenness centrality, the USA ranked first (0.36), followed by Italy (0.22) and China (0.15), indicating their pivotal roles as hubs in international collaboration. The USA showed the highest centrality, which may be related to its early contributions to foundational studies on metabolic rescue, MSC-mediated mitochondria transfer, lung injury, and neuroprotection. China had the highest publication output, reflecting its rapid expansion in recent years, particularly in MSC/iPSC-MSC-mediated tissue repair, cardiovascular and neural injury, bone homeostasis, tumor biology, and immune regulation. However, China’s lower centrality than that of the USA suggests that its international bridging role remains to be further strengthened.

**Table 1:** Top countries/regions and institutions contributing to mitochondria transfer research.

Rank	Country/ regions	Year	Count	Centrality	Institutions	Year	Count	Centrality
1	China	2007	373	0.15	University of California System	2016	30	0.15
2	USA	2006	198	0.36	Institut National de la Sante et de la Recherche Medicale (Inserm)	2011	28	0.14
3	Japan	2010	56	0.06	Zhejiang University	2021	27	0.19
4	Italy	2010	49	0.22	Shanghai Jiao Tong University	2018	26	0.05
5	France	2011	37	0.07	sun yat-sen university	2018	25	0.04
6	Australia	2016	32	0.07	Harvard University	2016	23	0.2
7	England	2015	26	0.12	Nanjing Medical University	2019	21	0.13
8	South Korea	2012	25	0.02	Universidad de los Andes	2019	17	0.04
9	India	2014	24	0.02	Czech Academy of Sciences	2016	15	0
10	Germany	2012	24	0.06	Sichuan University	2024	15	0.02

At the institutional level (**Figure 3B** and **Table 1**), the most productive institutions were the University of California System (30 publications), Inserm (28), Zhejiang University (27), Shanghai Jiao Tong University (26), and Sun Yat-sen University (25). Harvard University showed the highest centrality (0.20), followed by Zhejiang University (0.19), the University of California System (0.15), Inserm (0.14), and Nanjing Medical University (0.13). Notably, the bright red nodes in the institutional network indicate that the publications of these institutions were concentrated in more recent years, suggesting relatively high research activity in the most recent phase of the field. In particular, several Chinese institutions entered this field relatively recently, such as Zhejiang University in 2021 and Sichuan University in 2024, indicating the rapidly increasing contribution of Chinese institutions to this area in recent years.

Overall, research on mitochondria transfer is characterized by expanding international collaboration, with China and the USA serving as the major contributors, while Chinese research institutions have emerged as increasingly active and important participants in recent years.

### 3.3 Author analysis of mitochondria transfer research

**Figure 4:**
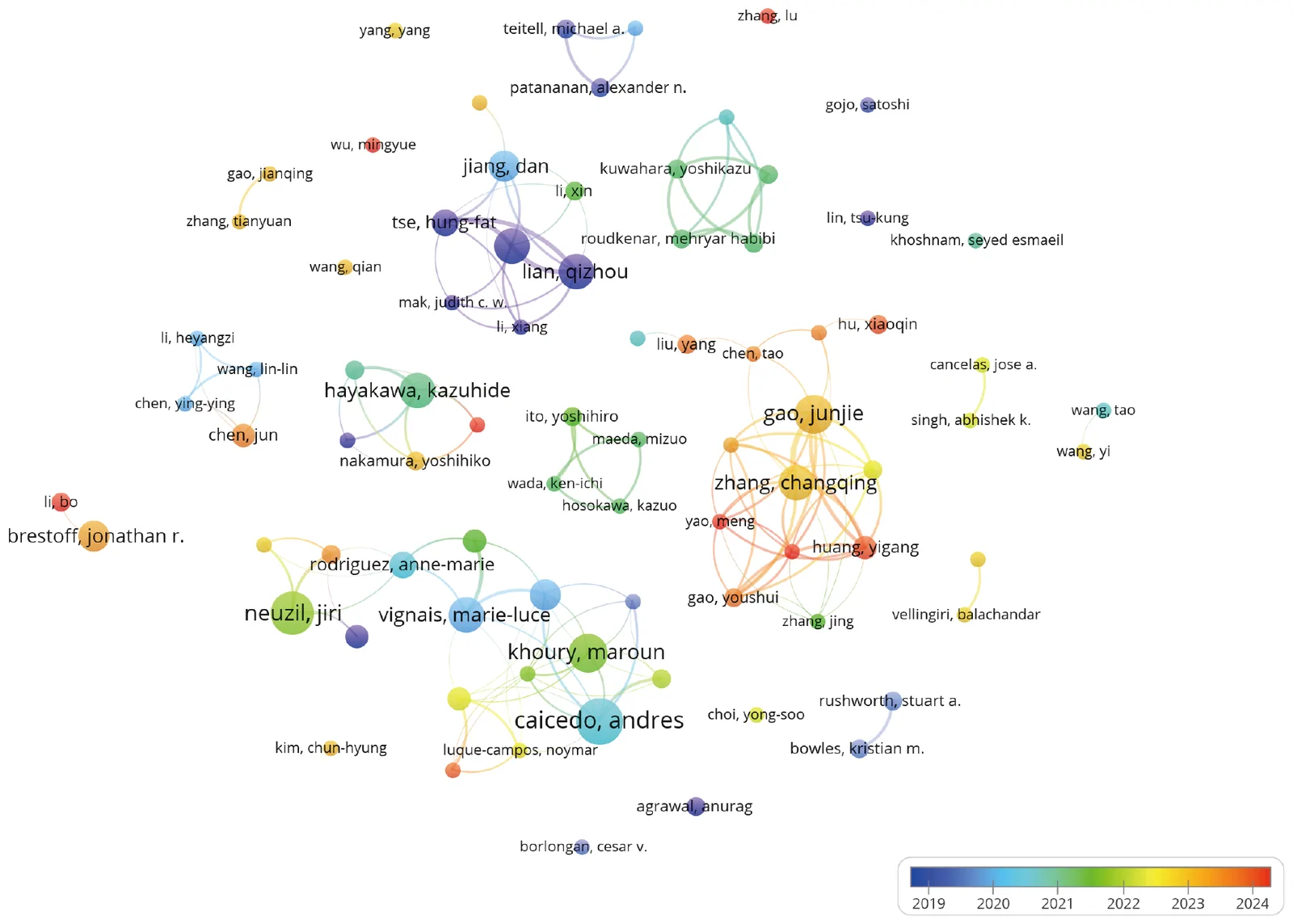
Visualization of the author collaboration network in mitochondria transfer research. Node size represents the publication output of each author, links indicate co-authorship relationships, and colors reflect the average publication year. Parameters: unit of analysis = authors; minimum documents per author = 4; minimum citations per author = 0; 82 of 5,273 authors met the thresholds; normalization = association strength; attraction = 2; repulsion = −5; clustering resolution = 1.00; minimum cluster size = 1; small clusters were merged.

As shown in **Figure 4** and **Table 2**, the author collaboration network in mitochondria transfer research consisted of several relatively distinct clusters, indicating that multiple active research groups have emerged in this field. Caicedo, Andres, was the most prolific author, with 12 publications, followed by Neuzil, Jiri (11); and Gao, Junjie; and Khoury, Maroun (10 each). In terms of citation impact, Zhang, Yuelin ranked first with 2,477 citations, followed by Lian, Qizhou (2,046), and Hayakawa, Kazuhide (1,523). Notably, Gao, Junjie; Zhang, Changqing; Zhang, Yuelin; and Lian, Qizhou showed relatively high total link strength, indicating strong collaborative connections within the co-authorship network. In addition, the warmer-colored nodes, particularly those associated with the Gao, Junjie–Zhang, and Changqing cluster, suggest that these authors have shown relatively high research activity in recent years. Further analysis showed that different author clusters corresponded to distinct research directions. The Caicedo-related cluster mainly focused on artificial mitochondria transfer and MitoCeption. The Neuzil-related cluster was associated with mitochondria transfer in cancer metabolism and tumor progression. The Gao, Junjie–Zhang, Changqing cluster focused on mitochondria transfer in bone homeostasis and musculoskeletal repair. The Zhang, Yuelin–Lian, Qizhou cluster was mainly related to iPSC-MSC–mediated mitochondrial transfer and cardiomyocyte protection. The Khoury-related cluster contributed to MSC-mediated immune regulation, whereas Hayakawa-related studies focused on astrocyte-to-neuron mitochondria transfer and neuroprotection.

**Table 2:** Top authors contributing to mitochondria transfer research.

Rank	Author	Institution (country)	Number	Citations	Total link strength
1	Caicedo, Andres	Universidad San Francisco de Quito (Ecuador)	12	790	23
2	Neuzil, Jiri	Czech Academy of Sciences (Czech Republic)	11	474	14
3	Gao, Junjie	Shanghai Sixth People’s Hospital (China)	10	623	42
4	Khoury, Maroun	Universidad de los Andes (Chile)	10	591	14
5	Hayakawa, Kazuhide	Massachusetts General Hospital (USA)	9	1523	16
6	Lian, Qizhou	University of Hong Kong (China)	9	2046	27
7	Vignais, Marie-Luce	University of Montpellier (France)	9	766	18
8	Zhang, Changqing	Shanghai Jiao Tong University School of Medicine (China)	9	597	40
9	Zhang, Yuelin	Southern Medical University (China)	9	2477	28
10	Brestoff, Jonathan R.	Washington University School of Medicine in St. Louis (USA)	8	857	1

However, most authors exhibited relatively low total link strength, indicating that the overall co-authorship network remains fragmented and that broader, more stable intergroup collaborations have yet to be fully established. This fragmented pattern suggests that mitochondria transfer research has expanded into several specialized communities, including artificial mitochondrial transfer, tumor metabolism, MSC-mediated tissue repair, immune regulation, bone repair, and neuroprotection. Although this reflects the interdisciplinary nature of the field, it also indicates insufficient integration among different research communities. Collectively, these findings indicate that mitochondria transfer research has developed several influential author groups, although greater collaborative integration across the field is still needed.

### 3.4 Journal output analysis of mitochondrial transfer research

**Figure 5:**
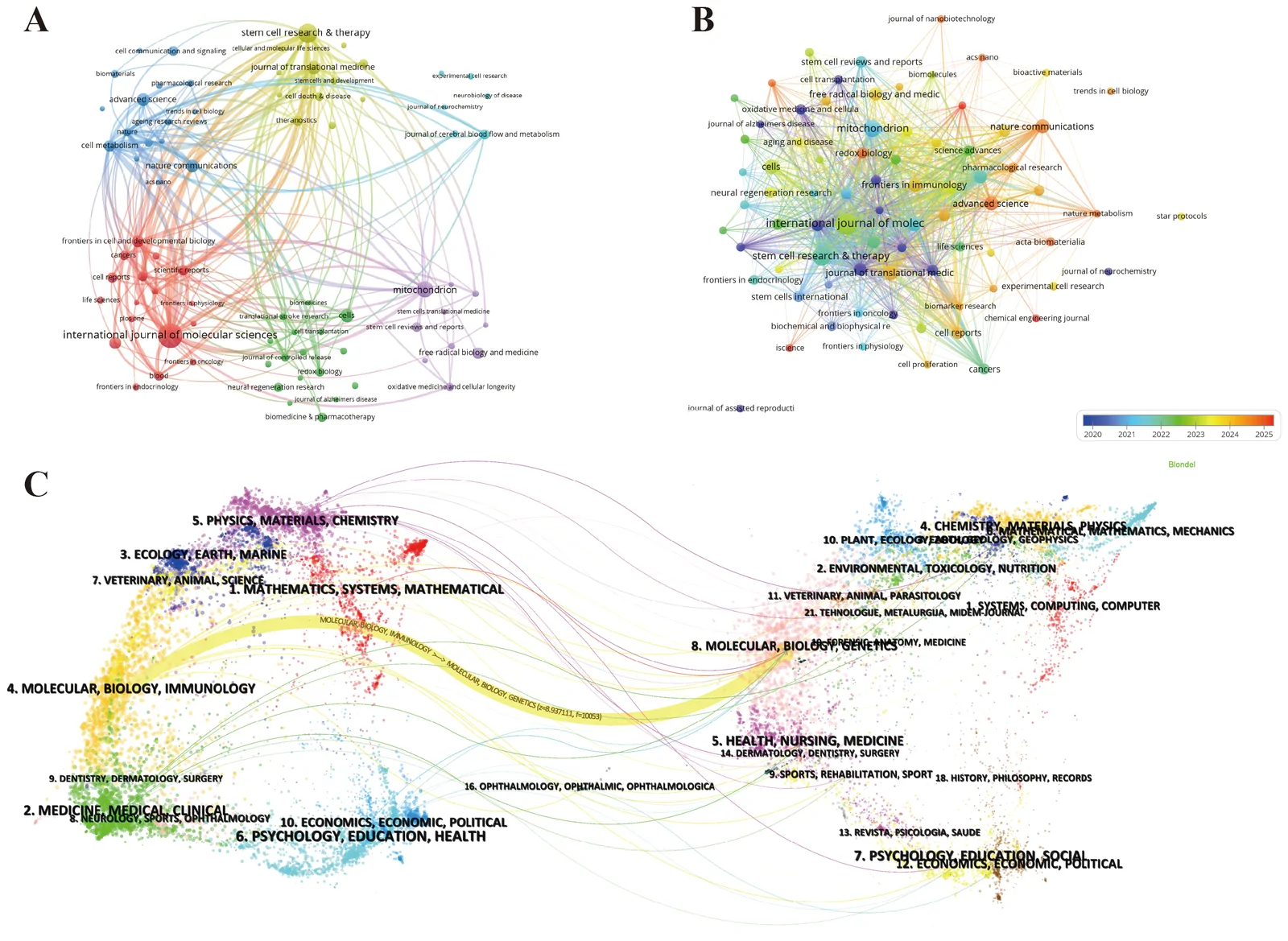
Journal visualization and dual-map overlay analysis of mitochondria transfer research. **(A)** Clustered network visualization of journals. **(B)** Overlay visualization of journals. Node size represents the number of publications of each journal, and links indicate relationships among journals. Colors in **(A)** represent clusters, whereas colors in **(B)** indicate the average publication year. Parameters: unit of analysis = sources; minimum documents per source = 3; minimum citations per source = 0; 78 of 374 sources met the thresholds; normalization = association strength; attraction = 3; repulsion = 0; clustering resolution = 1.00; minimum cluster size = 1; small clusters were merged. **(C)** Dual-map overlay of journals. The left side represents citing journals, the right side represents cited journals, and the colored curves indicate the major citation paths. Parameters: journal labels = 8, arcs = 3, citing articles ≥ 10, cited times ≥ 100, snap to centroids (<radius>) = 0, and *Z*-scores enabled.

As shown in **Figure 5A** and **Table 3**, the journal network of mitochondrial transfer research formed multiple interconnected clusters, indicating that this field spans diverse disciplines, including molecular and cellular biology, stem cell research, neuroscience, metabolism, biomaterials, and translational medicine. In terms of publication output, the International Journal of Molecular Sciences ranked first with 39 publications, followed by Stem Cell Research & Therapy (26) and Mitochondrion (21). With respect to citation performance, Stem Cell Research & Therapy (1,731 citations) and Cell Metabolism (1,694 citations) showed the greatest citation impact. In addition, the International Journal of Molecular Sciences exhibited the highest total link strength (411), followed by Stem Cell Research & Therapy (387), the Journal of Translational Medicine (320), and Frontiers in Cell and Developmental Biology (302), indicating that these journals occupy central positions within the journal network. The journal distribution reflects the evolution of mitochondria transfer research from early studies of mitochondrial biology, cellular metabolism, and stem cell repair to its recent expansion into immunology, redox biology, biomaterials, nanobiotechnology, and translational medicine. This trend indicates a shift from basic cell-biological mechanisms toward therapeutic and engineering-oriented applications.

**Table 3:** Top journals contributing to mitochondria transfer research.

Rank	Journal	Number	Citations	Total link strength	IF (2024)/JCR
1	International Journal of Molecular Sciences	39	1214	411	4.9/Q1
2	Stem Cell Research & Therapy	26	1731	387	7.3/Q1
3	Mitochondrion	21	535	218	4.5/Q2
4	Cells	14	393	105	5.2/Q2
5	Journal of Translational Medicine	14	381	320	7.5/Q1
6	Advanced Science	13	254	112	14.1/Q1
7	Nature Communications	13	268	127	15.7/Q1
8	Frontiers in Cell and Developmental Biology	11	740	302	4.3/Q1
9	Frontiers in Immunology	11	220	89	5.9/Q1
10	Cell Metabolism	10	1694	277	30.9/Q1

As shown in **Figure 5B**, the publication venues of mitochondria transfer research exhibited a clear temporal evolution. Early studies were mainly published in journals related to mitochondrial biology, stem cell research, and cellular metabolism, such as Mitochondrion, Stem Cell Research & Therapy, and Cell Metabolism. Subsequently, the field expanded to include journals emphasizing molecular mechanisms and immune regulation, including the International Journal of Molecular Sciences, Cells, Frontiers in Immunology, and Free Radical Biology and Medicine. In recent years, related studies have increasingly appeared in the Journal of Translational Medicine, Advanced Science, Nature Communications, Redox Biology, Biomaterials, Journal of Nanobiotechnology, and Nature Metabolism, indicating a progressive shift toward translational medicine and interdisciplinary applications. The dual-map overlay in **Figure 5C** further supported this trend, with the principal citation pathway extending from Molecular/Biology/Immunology to Molecular/Biology/Genetics. This pattern suggests that the field remains grounded in molecular biology and genetics while expanding toward translational and interdisciplinary research.

Overall, these findings indicate that mitochondria transfer research is distributed across multidisciplinary journals and is showing a sustained trend toward translational research and emerging interdisciplinary frontiers.

### 3.5 Co-cited reference analysis of mitochondria transfer research

The co-cited reference network of mitochondria transfer research revealed a well-defined knowledge structure centered on foundational mechanistic studies, with subsequent expansion toward disease-related applications and translational topics. First, based on the highly cited references listed in **Table 4**, the studies most closely related to mitochondria transfer included Spees et al. (2006), Islam et al. (2012), Ahmad et al. (2014), and Hayakawa et al. (2016) (10–12, 27). These studies directly addressed the phenomenon of intercellular mitochondria transfer, its key regulatory mechanisms, and its protective roles in lung injury and stroke, thereby establishing the conceptual and mechanistic foundation of the field. Subsequent studies, including Jackson et al. (2016), Morrison et al. (2017), and Moschoi et al. (2016) (28–30), further extended this field to tunneling nanotube-mediated transfer, extracellular vesicle-mediated transfer, and tumor and leukemia microenvironments, reflecting the progression of mechanistic insights into disease-oriented contexts. By contrast, review articles such as Liang et al. (2014), Spees et al. (2016), and Fan et al. (2020) (31–33) summarized the role of mitochondria transfer mainly from the perspective of MSC-mediated reparative mechanisms. Although these reviews were less directly related to the core mechanistic evidence than the original studies, they remained important for knowledge synthesis and conceptual integration.

**Table 4:** Top globally cited references in mitochondria transfer research.

Rank	Citations	First author	Year	Journal	Tittle	Type of paper
1	1344	Islam MN	2012	Nature medicine	Mitochondrial transfer from bone marrow–derived stromal cells to pulmonary alveoli protects against acute lung injury	Article
2	1176	Hayakawa K	2016	Nature	Transfer of mitochondria from astrocytes to neurons after stroke	Article
3	957	Spees JL	2006	PNAS	Mitochondrial transfer between cells can rescue aerobic respiration	Article
4	805	Liang XT	2014	Cell transplantation	Paracrine mechanisms of mesenchymal stem cell–based therapy: current status and perspectives	Review
5	726	Spees JL	2016	Stem cell research & therapy	Mechanisms of mesenchymal stem/stromal cell function	Review
6	680	Morrison TJ	2017	Am. J. Respir. Crit. Care Med.	Mesenchymal stromal cells modulate macrophages in clinically relevant lung injury models by extracellular vesicle mitochondrial transfer	Article
7	543	Ahmad T	2014	The EMBO Journal	Miro1 regulates intercellular mitochondrial transport & enhances mesenchymal stem cell rescue efficacy	Article
8	481	Jackson MV	2016	Stem Cells	Mitochondrial transfer via tunneling nanotubes is an important mechanism by which mesenchymal stem cells enhance macrophage phagocytosis in the *in-vitro* and *in-vivo* models of ARDS	Article
9	456	Fan XL	2020	Cellular and Molecular Life Sciences	Mechanisms underlying the protective effects of mesenchymal stem cell–based therapy	Review
10	379	Moschoi R	2016	Blood	Protective mitochondrial transfer from bone marrow stromal cells to acute myeloid leukemic cells during chemotherapy	Article

**Figure 6:**
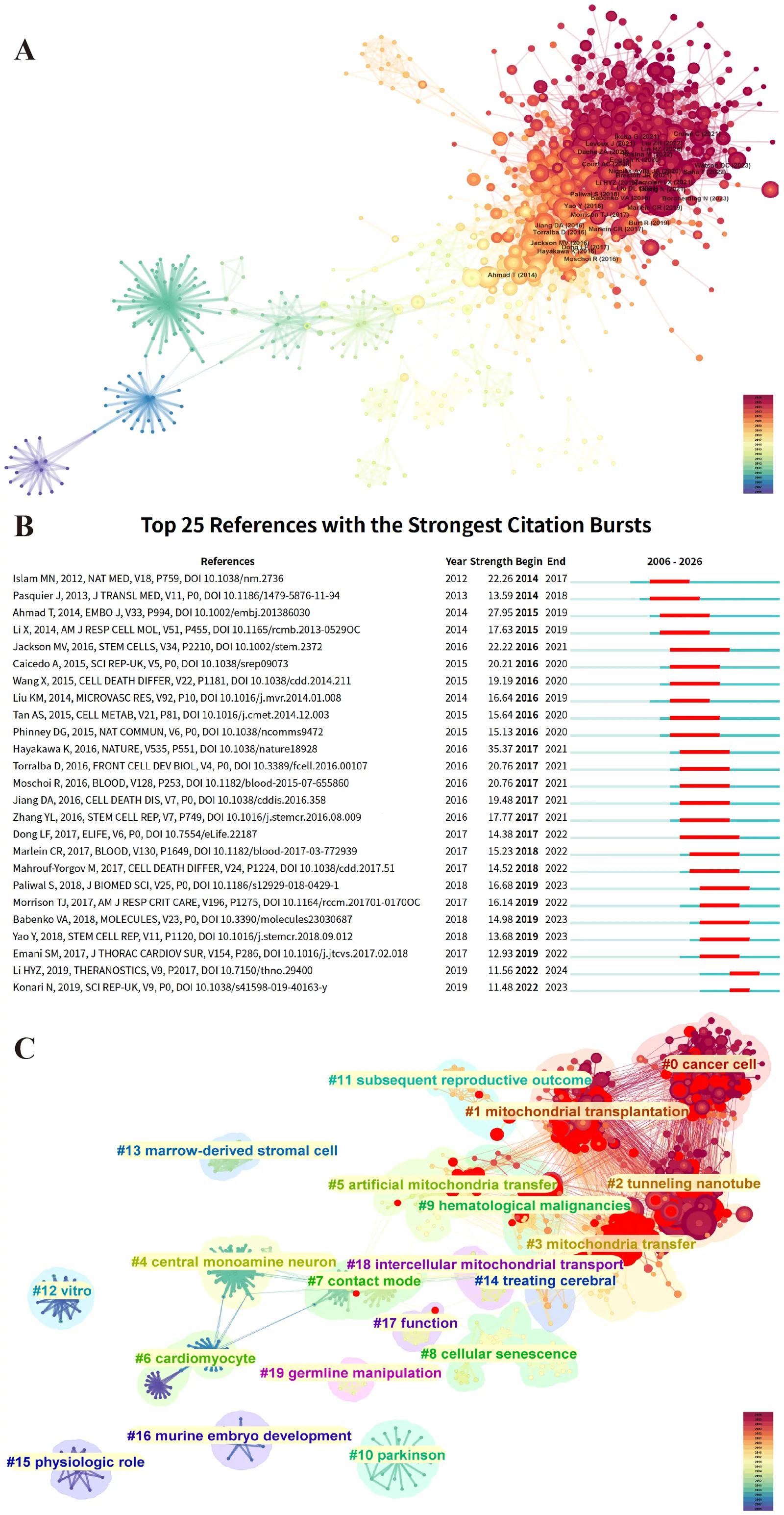
Co-cited reference analysis in mitochondria transfer research. **(A)** Co-cited reference network. **(B)** Top 25 references with the strongest citation bursts. **(C)** Timeline view of co-cited reference clusters. Parameters: CiteSpace (v) 7.0.R0; time span = 2006–2026; slice length = 1 year; node type = cited reference; selection criteria = g-index (*k* = 25), LRF = 2.5, L/N = 10, LBY = 5, e = 1.0; pruning = none. Citation/frequency burst periods were highlighted in tree rings. Burst detection parameters were d1/d0 = 2.0, αi/αi−1 = 2.0, number of states = 2, γ = 1.0, and minimum duration = 2 years. A total of 213 burst items were detected, and the top 25 references are shown in **(B)**. Cluster labels in **(C)** were generated from title terms using the LLR algorithm, with the top 20 continuous clusters displayed along the timeline. Modularity Q = 0.6606 and weighted mean silhouette S = 0.7688.

Second, when considered together with **Figures 6A, B**, and the temporal distribution of burst references, mitochondria transfer research appeared to evolve from conceptual establishment to mechanistic expansion and subsequently to increasingly disease-oriented applications. Early burst references were mainly associated with the recognition of the biological significance of mitochondria transfer and the identification of key regulatory factors, indicating that the initial stage of the field focused primarily on the discovery of the phenomenon and mechanistic validation. This was followed by a shift in burst hotspots toward disease contexts such as stroke, leukemia, and lung injury, suggesting a transition from basic mechanistic studies to disease-oriented applications. In more recent years, research hotspots have increasingly focused on neural repair, the tumor microenvironment, and clinical translation, indicating that the field has gradually progressed from fundamental observations toward mechanistic refinement and translational application.

Finally, the co-cited reference title clusters shown in **Figure 6C** indicated that the topics most strongly associated with mitochondria transfer included #3 mitochondria transfer, #2 tunneling nanotubes, #1 mitochondrial transplantation, #5 artificial mitochondria transfer, and #18 intercellular mitochondrial transport. Together, these clusters constituted the core knowledge framework of the field. Clusters with moderate relevance mainly included #0 cancer cell, #9 hematological malignancies, #13 marrow-derived stromal cell, and #14 treating cerebral, reflecting the principal application areas of tumor biology, hematological diseases, and neural injury. By contrast, clusters related to reproduction, embryonic development, specific organ contexts, and nervous system disease models occupied a relatively peripheral position. Taken together, mitochondria transfer research has evolved from an early stage of basic mechanistic exploration into a research system with both disease-oriented significance and translational potential. Tunneling nanotubes, mitochondrial transplantation, cancer cells, and hematological malignancies represent some of the most central and active research frontiers in this field.

### 3.6 Keyword analysis of mitochondria transfer research

**Figure 7:**
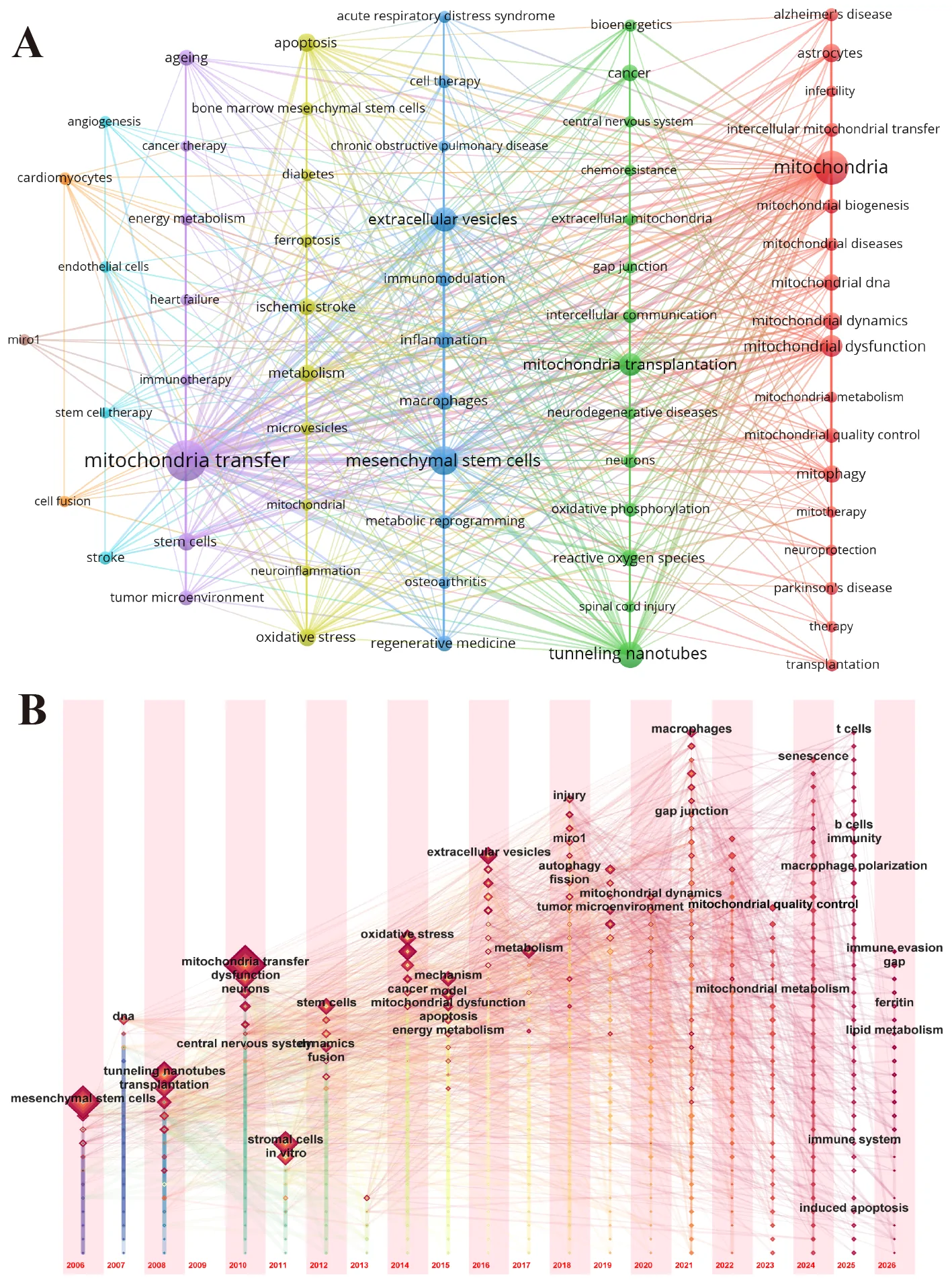
Keyword co-occurrence network and temporal evolution in mitochondria transfer research. **(A)** Keyword co-occurrence network. Node size represents keyword frequency, links indicate co-occurrence relationships, and colors represent different clusters. The network was generated using VOSviewer and further optimized in Pajek for layout adjustment. Parameters: minimum keyword occurrences = 6; 68 of 1,653 keywords met the threshold; clustering resolution = 1.00; minimum cluster size = 1; small clusters were merged. **(B)** Keyword timezone view. The map was generated using CiteSpace (v) 7.0.R0. Parameters: time span = 2006–2026; slice length = 1 year; node type = keyword; selection criteria = g-index (k = 25), LRF = 2.5, L/N = 10, LBY = 5, e = 1.0; pruning = none. No additional minimum occurrence threshold was applied beyond the g-index-based selection criterion.

As shown in **Figure 7A**, the keyword co-occurrence network of mitochondria transfer research was organized into eight major clusters, mainly involving mitochondrial homeostasis and dysfunction, intercellular transfer mechanisms, stem cell–mediated repair, inflammatory and metabolic regulation, tumor biology, and neurological diseases. Core keywords included mitochondria, mitochondria transfer, mesenchymal stem cells, tunneling nanotubes, mitochondria transplantation, and extracellular vesicles, indicating that these topics constitute the principal conceptual and mechanistic framework of the field. Specifically, the major clusters were characterized by themes related to mitochondrial dysfunction and quality control (Cluster 1), transfer mechanisms and disease applications (Cluster 2), stem cell–mediated repair and immunomodulation (Cluster 3), stress injury and cell death mechanisms (Cluster 4), disease and therapeutic contexts of mitochondria transfer (Cluster 5), vascular repair and regenerative therapy (Cluster 6), cardiomyocyte-related studies (Cluster 7), and the Miro1-associated regulatory mechanisms (Cluster 8).

As a complement to the VOSviewer clustering results, the CiteSpace keyword timezone view (**Figure 7B**) further revealed the temporal evolution of this field. Early studies mainly focused on mesenchymal stem cells, tunneling nanotubes, transplantation, neurons, the central nervous system, and mitochondria transfer, whereas later research increasingly expanded toward mitochondrial dysfunction, oxidative stress, extracellular vesicles, the tumor microenvironment, and metabolism. In more recent years, keywords such as Miro1, autophagy, gap junction, macrophages, mitochondrial dynamics, mitochondrial quality control, senescence, T cells, B cells, immunity, macrophage polarization, and immune evasion have become more prominent, indicating that current studies are shifting toward more refined regulatory mechanisms, immune–metabolic interactions, and disease-oriented translational applications. Overall, these findings suggest that mitochondria transfer research has evolved from early studies of basic transfer phenomena and stem cell–mediated rescue toward a broader research framework involving mitochondrial quality control, tumor and immune microenvironments, and translational therapeutic strategies.

## 4 Discussion

### 4.1 General information in mitochondria transfer research

Based on 851 publications related to mitochondria transfer retrieved from 2006 to 8 April 2026, this study systematically revealed the rapid growth and multidisciplinary integration of this field. The included publications comprised 566 original research articles and 285 reviews, with 38,879 total citations and an average of 45.69 citations per publication. Annual publication output remained generally low before 2015, increased markedly after 2018, and reached its highest level among complete calendar years in 2025. It should be noted that the 2026 dataset included only publications indexed up to 8 April 2026 and therefore represented partial-year data. Accordingly, the 2026 results were interpreted as reference data reflecting the most recent stage of the field. Overall, these findings suggest that mitochondria transfer research has moved from early identification of the phenomenon and mechanistic exploration into a period of rapid development.

From the perspective of the research landscape, China and the USA were the leading contributors, while the USA, Italy, and China showed relatively high betweenness centrality in the collaboration network. The University of California System, Inserm, and several Chinese universities made prominent contributions; however, the author collaboration network remained relatively fragmented, indicating that collaboration across teams and regions still needs to be strengthened. Journal distribution showed that mitochondria transfer research has gradually expanded from basic fields such as mitochondrial biology, stem cell research, and cellular metabolism to multidisciplinary directions, including immune regulation, biomaterials, redox biology, and translational medicine. This indicates that the field is progressively extending from basic mechanistic research toward the investigation of disease mechanisms and translational applications.

### 4.2 Research hotspots and trends in mitochondria transfer research

The co-cited title clustering results suggest that mitochondria transfer research is characterized by the diversity of transfer routes and associated disease contexts. In this study, title clusters closely related to mitochondria transfer included tunneling nanotubes, artificial and intercellular mitochondria transfer, mitochondrial transplantation, and related topics, which together constitute the core knowledge framework of this field. Existing studies have shown that various cell types, acting as donor cells, can export and deliver their mitochondria to other cell types, referred to as recipient cells. This transfer can occur through either contact-dependent routes, such as tunneling nanotubes, gap junctions, and dendritic structure-mediated transfer, or non-contact-dependent routes, such as extracellular vesicles carrying mitochondria and extracellular mitochondria (9, 34). Different transfer routes are regulated by relatively specific but partially overlapping molecular mechanisms. Among these mechanisms, dendritic structure-mediated mitochondria transfer between osteocytes requires MFN2 and VAPB (34, 35). Gap junction-mediated mitochondria transfer is mainly dependent on connexin 43 (Cx43), which may also participate in mitochondria transfer mediated by TNTs and extracellular vesicles (11, 36, 37). Extracellular vesicle-mediated mitochondria transfer, commonly characterized by vesicular markers such as CD63, CD9, and CD81, represents another frequently reported mode of mitochondria transfer and may be influenced by pathways mediated by PINK1–Parkin and CD38 (7, 38). M-Sec, small GTPases, Cdc42, the exocyst complex, leukocyte-specific transcript 1, and GAP43 are involved in the formation and elongation of TNTs, with F-actin and microtubules serving as their structural scaffold. Mitochondrial trafficking through TNTs is one of the most extensively described transfer routes and requires mitochondrial outer membrane-associated proteins such as Miro1 and Myo19 (7, 34, 39, 40). However, the key molecules and precise mechanisms underlying these transfer routes remain incompletely defined, which may represent an important focus of future research. In addition, clusters such as cancer cells, hematological malignancies, marrow-derived stromal cells, and treating cerebral also suggest that mitochondria transfer is closely associated with stem cell–mediated repair, neurological injury, hematological malignancies, cancer, and related disease contexts.

**Figure 8:**
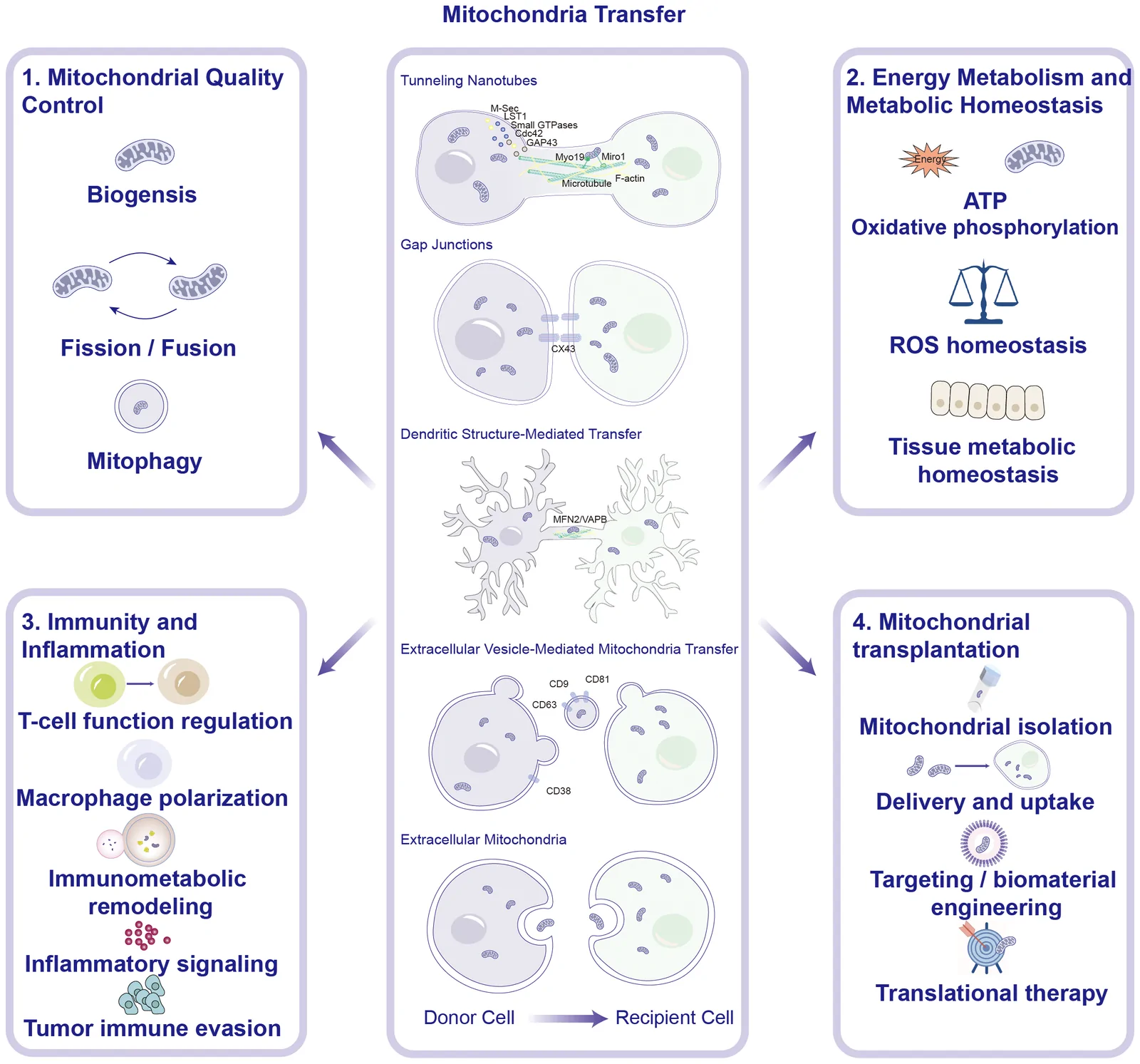
Schematic overview of the mechanisms of mitochondria transfer.

Building on these diverse transfer routes and their molecular regulation, keyword clustering and timeline analyses further revealed the evolution of research hotspots in mitochondria transfer. The emergence of themes such as mitochondrial dysfunction and quality control, inflammation and metabolic regulation, Miro1-related mechanisms, macrophages, T cells, B cells, immune evasion, mitochondrial dynamics, and autophagy indicates that this field is increasingly shifting toward the regulation of cellular homeostasis, metabolic remodeling, and microenvironmental remodeling. Together, these findings support an integrated view that mitochondria transfer represents an important mechanism of immunometabolic communication, linking mitochondrial quality control, metabolic remodeling, immune regulation, and disease progression. After transfer into recipient cells, exogenous mitochondria may interact with or be integrated into endogenous mitochondrial networks, be selectively cleared through mitophagy, or act as mitochondrial stress signals, thereby reshaping mitochondrial quality control in recipient cells (41–43). These changes further influence oxidative phosphorylation, ATP production, redox balance, mtROS generation, and metabolic flexibility. These processes are key determinants of immune-cell activation, macrophage polarization, T-cell fitness, inflammatory signaling, and tissue microenvironment remodeling (29, 41, 44, 45). The transfer of functional mitochondria can restore mitochondrial homeostasis and promote anti-inflammatory or tissue-protective immune responses (29, 42, 44). Conversely, in pathological contexts such as obesity, chronic inflammation, or cancer, impaired mitophagy in donor cells or the transfer of damaged or mutation-bearing mitochondria may amplify mitochondrial stress and disrupt immune cell metabolism. These effects may promote inflammatory injury, immune dysfunction, exhaustion, or immune evasion (41, 45–48). Thus, the outcome of mitochondria transfer is determined not only by the occurrence of mitochondrial exchange itself but also by the quality control status of transferred mitochondria, the metabolic state of recipient cells, and the local immune–inflammatory microenvironment. Accordingly, the following discussion focuses on four major frontiers: mitochondrial quality control, energy metabolism and other aspects of metabolic homeostasis, immunity and inflammation, and mitochondrial transplantation (**Figure 8**).

#### 4.2.1 Mitochondrial quality control

Bibliometric findings indicate that mitochondrial quality control has become an important mechanistic theme in mitochondria transfer research. Keywords related to mitochondrial dysfunction, mitochondrial dynamics, mitophagy, oxidative stress, autophagy, and senescence were closely associated with mitochondria transfer. These keywords were also linked to mesenchymal stem cells, tunneling nanotubes, and extracellular vesicles, indicating a shift from describing transfer events toward understanding how mitochondrial fitness and turnover influence their functional consequences. Multiple studies have shown that mitochondrial transfer is closely associated with mitochondrial quality control processes, including mitochondrial biogenesis, mitochondrial fission and fusion, and mitophagy (7, 49, 50). These quality control processes influence the quantity, structural integrity, and functional status of transferable mitochondria in donor cells, as well as the integration, clearance, and functional consequences of transferred mitochondria in recipient cells.

Mitochondrial biogenesis maintains mitochondrial quantity and function by generating new mitochondrial components and expanding pre-existing mitochondrial networks (51). Current evidence suggests that this process is associated with intercellular mitochondria transfer. Melanoma cells can induce PGC-1α upregulation in bone marrow–derived stromal cells, thereby promoting mitochondrial biogenesis in donor cells and providing a basis for subsequent mitochondrial transfer to melanoma cells through direct cell contact (52). In pulmonary fibrosis, pharmacological and nanomaterial-based enhancement of mitochondrial biogenesis in donor cells increased mitochondrial transfer and improved ATP production, respiratory function, and tissue repair in recipient cells (53). MoS_2_ nanomaterials also enhanced mitochondrial biogenesis in donor cells and increased mitochondria transfer efficiency (54). In recipient cells, mitochondria-rich extracellular vesicles delivered functional mitochondria and promoted mitochondrial biogenesis through mitochondrial genome transfer (55). These findings suggest a bidirectional association between mitochondrial biogenesis and mitochondria transfer, although the underlying regulatory mechanisms remain incompletely understood.

Mitochondrial fission and fusion are also associated with the efficiency and functional outcomes of mitochondria transfer. In donor cells, appropriate mitochondrial fission may facilitate the separation of mitochondria from the original mitochondrial network and their subsequent transfer to recipient cells. Fragmented mitochondrial networks in pro-tumorigenic macrophages were associated with increased mitochondria transfer, and the transferred mitochondria promoted ROS accumulation, ERK activation, and tumor-cell proliferation (56). In hepatocellular carcinoma, MTFR2-mediated mitochondrial fission in hepatic stellate cells promoted the co-transfer of fatty acids and mitochondria to tumor cells, supporting metabolic adaptation and tumor progression (57). Fission associated with Drp1 and Mff promoted macrophage-derived mitochondria transfer and improved neurovascularization in diabetic bone regeneration (58). Drp1-mediated fission also enhanced mitochondria transfer from macrophages to bone marrow mesenchymal stem cells during mechanical force-induced bone formation (59). After transfer, mitochondrial fusion may facilitate the interaction of exogenous mitochondria with the endogenous mitochondrial network. Live-cell transplantation studies showed that transferred mitochondria rapidly fused with recipient-cell mitochondria (60). Similarly, mesenchymal stem cell–derived mitochondria fused with the damaged mitochondrial network of neurons in a spinal cord injury model, accompanied by improved mitochondrial function and reduced neuronal ferroptosis (61). In contrast, macrophage-derived mitochondria-rich extracellular vesicles disrupted mitochondrial dynamics in recipient bone marrow mesenchymal stem cells through signaling involving LCN2, OMA1, and OPA1 and aggravated inflammatory bone loss (62). Moreover, RIPK4-mediated MFN2 degradation induced mitochondrial fragmentation and inhibited mitochondrial transfer (63), indicating that proteins involved in mitochondrial dynamics may regulate both transfer efficiency and its functional consequences.

Mitophagy and mitochondrial transfer jointly contribute to mitochondrial quality control. In donor cells, mitophagy affects the quality of mitochondria available for transfer. Obesity reduced cardiolipin levels, LC3-dependent autophagosome formation, and mitophagy in mesenchymal stem cells, resulting in the accumulation of damaged mitochondria and impaired transfer of functional mitochondria to airway epithelial cells. Pharmacological restoration of mitophagy can improve mitochondrial transfer and therapeutic efficacy (41). In recipient cells, transferred mitochondria may also regulate mitochondrial homeostasis by activating mitophagy. In endothelial cells, internalized mitochondria triggered PINK1–Parkin-dependent mitophagy, which was required for the enhanced engraftment capacity of mitochondria-transplanted endothelial cells (42). In pulmonary fibrosis, enhanced mitochondrial transfer restored mitophagy and mitochondrial homeostasis in recipient cells (53). In contrast, tumor-derived mitochondria carrying mutant mtDNA can resist autophagic clearance after transfer into tumor-infiltrating T cells, leading to metabolic dysfunction, senescence, and impaired T-cell function (45). In gastric cancer, mesenchymal stem cell–derived mitochondria fused with the mitochondrial network of cancer cells and reduced mitophagy, thereby promoting cell survival (64). Notably, mitochondrial transfer can also function as a form of transcellular mitophagy known as transmitophagy, whereby cone photoreceptors transfer damaged mitochondria to Müller glial cells for processing under stress conditions (65). These findings indicate that mitophagy influences both the quality of transferred mitochondria and their subsequent fate in recipient cells.

Mitochondrial quality control may influence the immunometabolic effects mediated by intercellular mitochondria transfer. In donor cells, impaired mitophagy can lead to the accumulation of dysfunctional mitochondria and reduce the transfer of functional organelles. Obesity impairs mitophagy dependent on cardiolipin and LC3 in MSCs, thereby reducing mitochondria transfer to airway epithelial cells and weakening the associated anti-inflammatory effects. Restoration of mitophagy can improve both mitochondria transfer and therapeutic efficacy (41). Similarly, extracellular vesicle-mediated mitochondria transfer from MSCs to macrophages enhances macrophage oxidative phosphorylation and phagocytic activity. When mitochondrial function is impaired in donor cells, these immunomodulatory effects are markedly reduced, suggesting that the functional status of donor mitochondria may influence the biological consequences of mitochondria transfer (29). Mitochondrial quality control responses in recipient cells are also important. Under high glucose conditions, mitochondria transfer from MSCs can activate mitochondrial biogenesis through PGC-1α and lysosomal autophagy through the PGC-1α/TFEB pathway in macrophages, thereby promoting the clearance of damaged mitochondria and macrophage polarization toward an anti-inflammatory phenotype. These effects are substantially attenuated by PGC-1α knockdown or impairment of donor mitochondria (66). In addition, MSCs can export partially depolarized mitochondria through extracellular vesicles, which are subsequently taken up and reutilized by macrophages, suggesting a potential form of transcellular mitochondrial quality control (67). Conversely, mitochondria derived from tumor cells and carrying mtDNA mutations can evade mitophagic clearance after transfer into T cells, thereby promoting tumor immune evasion (45). Collectively, these studies indicate that both the functional status of donor mitochondria and the capacity of recipient cells to utilize or eliminate transferred mitochondria can influence metabolic adaptation and immune function. Mitochondrial quality control may therefore represent an important determinant of immunometabolic communication mediated by mitochondrial transfer.

#### 4.2.2 Regulation of energy metabolism and metabolic homeostasis

Keyword analysis identified metabolic regulation as a major theme in mitochondria transfer research. Energy metabolism, oxidative phosphorylation, bioenergetics, mitochondrial dysfunction, oxidative stress, and reactive oxygen species were closely connected in the co-occurrence network. The timezone analysis further showed increasing attention to metabolism, the tumor microenvironment, mitochondrial dynamics, mitochondrial quality control, and lipid metabolism. These findings indicate that the focus of mitochondria transfer research has expanded from bioenergetic rescue to metabolic remodeling and tissue homeostasis.

Abnormal energy metabolism is widely implicated in the occurrence and progression of various diseases and is often closely associated with mitochondrial dysfunction, metabolic disorders, and impaired cellular function (68). As mitochondria are the core organelles of energy metabolism, the most direct effect of their intercellular horizontal transfer is the delivery of respiration-competent mitochondria to recipient cells with mitochondrial dysfunction or energetic impairment, thereby restoring ATP production, oxygen consumption capacity, and cellular functional activity. Related studies have shown that exogenous mitochondrial delivery can improve mitochondrial membrane potential, oxygen consumption rate, and ATP production in recipient cells (69). Wharton’s jelly-derived MSCs can transfer mitochondria to cells with mitochondrial defects and restore impaired oxidative phosphorylation and bioenergetic function (70). In models of diabetes-related myocardial injury and myocardial ischemia, mitochondria transfer or mitochondria-rich extracellular vesicles can improve cardiomyocyte bioenergetic function and restore myocardial energy metabolism (71, 72). During stress erythropoiesis, erythroblastic island macrophages transfer mitochondria to early erythroblasts and alter their bioenergetic profile through CD47, thereby promoting erythroid recovery after anemic stress (73). Similarly, in osteoarthritic chondrocytes, MSC-derived mitochondria transfer can increase ATP production and the OXPHOS-to-glycolysis ratio (74). Together, these studies indicate that mitochondria transfer restores the bioenergetic capacity of recipient cells by restoring functional mitochondrial networks. In the tumor microenvironment, this energy-compensatory effect can also be exploited by tumor cells. For example, cancer-associated fibroblasts, bone marrow stromal cells, platelets, and neural cells can transfer mitochondria to tumor cells, thereby enhancing oxidative phosphorylation, ATP production, energy metabolism, survival, and related malignant phenotypes (75–79). Interestingly, transplantation of astrocytic mitochondria enhanced aerobic respiration in glioma cells, reduced glycolysis, and inhibited malignant proliferation (80).

Beyond its role in basic energy metabolism, mitochondria transfer also regulates metabolic homeostasis. In osteoarthritic chondrocytes, MSC-derived mitochondria increased SOD2 expression and activity, reduced ROS accumulation, and improved mitochondrial dynamics, thereby coordinating bioenergetic recovery with redox homeostasis (74). Glucose and lactate metabolism can also regulate the transfer process itself. In cerebral ischemia, astrocytic LRP1 reduced glucose uptake, glycolysis, and lactate production, thereby suppressing ARF1 lactylation and facilitating mitochondria transfer from astrocytes to neurons (81). Mitochondria transfer from MSCs can promote tip-cell transition after stroke through the regulation of glutathione metabolism, further demonstrating that its effects extend beyond direct energy supplementation (82).

Mitochondria transfer further links metabolic homeostasis to immunometabolic regulation by reshaping mitochondrial function in immune cells. In models of acute lung injury, MSCs transferred mitochondria to macrophages through TNTs or extracellular vesicles, thereby enhancing macrophage phagocytic capacity; extracellular vesicle-mediated transfer also increased macrophage oxidative phosphorylation (28, 29). Mitochondria transfer from adipocytes to macrophages can maintain metabolic homeostasis in white adipose tissue, whereas obesity or dietary lipids may disrupt this process and thereby affect both local adipose tissue metabolism and systemic energy homeostasis (46, 83). Platelet-derived mitochondria or mitochondria-associated extracellular vesicles can also alter the metabolic status of neutrophils and MSCs, thereby influencing inflammatory responses, immune homeostasis, and tissue repair (84, 85). These findings indicate that mitochondria transfer coordinates immune cell bioenergetics, mitochondrial turnover, and tissue adaptation, thereby linking immunometabolic regulation to tissue metabolic homeostasis. Its downstream effects on immune-cell phenotype and inflammatory signaling are discussed further in the following section.

Stressed or metabolically abnormal cells can expel mitochondria to reduce their burden of damaged mitochondria, while recipient cells further respond to or clear these mitochondria, thereby participating in the regulation of local or systemic metabolic homeostasis. Studies have shown that cardiomyocytes can expel dysfunctional mitochondria, which are subsequently cleared through phagocytosis by cardiac macrophages. This process helps prevent the accumulation of abnormal mitochondria and metabolic disturbances in the myocardium, thereby maintaining cardiac function (43). In adipose tissue, adipocyte-derived mitochondria can be taken up by macrophages to maintain metabolic homeostasis in white adipose tissue; however, this process is impaired under obese conditions (46). Dietary lipids can also inhibit macrophage uptake of adipocyte-derived mitochondria, causing these mitochondria to enter the circulation and be delivered to distant organs. This suggests that mitochondrial extrusion is not merely a form of waste disposal but may also participate in interorgan metabolic communication and adaptation to nutritional stress (83). Conversely, the transfer of damaged mitochondria can propagate metabolic injury. In osteoporosis, M1-like macrophages transferred oxidatively damaged mitochondria to MSCs, inducing excessive ROS production and succinate accumulation and thereby impairing osteogenic differentiation (86). Collectively, transfer of functional mitochondria generally supports cellular respiration, redox balance, and metabolic adaptability, whereas the transfer or inadequate clearance of damaged mitochondria can aggravate oxidative stress and pathological metabolic remodeling. Because immune cells directly participate in mitochondria transfer, uptake, and clearance, these metabolic exchanges also provide a mechanistic basis for the regulation of inflammatory signaling and immune cell function discussed in the next section.

#### 4.2.3 Immunity and inflammation

Bibliometric findings indicate that immunity and inflammation have become rapidly expanding themes in mitochondria transfer research. In the keyword co-occurrence and timezone analyses, inflammation, immunomodulation, macrophages, neuroinflammation, tumor microenvironment, T cells, B cells, macrophage polarization, senescence, and immune evasion were increasingly associated with mitochondria transfer and mesenchymal stem cells. These patterns suggest a shift from early studies of stem cell–mediated tissue repair toward immunometabolic communication, inflammatory microenvironment remodeling, and tumor immune regulation. Co-cited clusters such as #0 cancer cell and #9 hematological malignancies further indicate that tumor immunity and immune escape have emerged as important disease-oriented frontiers.

Mitochondria transfer is not merely a mechanism of metabolic compensation but also a context-dependent form of immunometabolic communication that connects mitochondrial quality, metabolic remodeling, immune cell function, and inflammatory signaling. Its immunological effects depend on the functional state of the transferred mitochondria, the identities of the donor and recipient cells, the transfer route, and the local inflammatory microenvironment. In adaptive immunity, T cells are among the most extensively studied recipient cells in immune regulation mediated by mitochondria transfer. Studies have shown that bone marrow–derived stromal cells can transfer mitochondria to T cells through TNTs, thereby enhancing T-cell metabolic fitness and antitumor efficacy (44). Similarly, mitochondria transfer from MSCs improves the survival of both native and engineered T cells, suggesting that this process may enhance the persistence of T cell-based therapies (87). During *Mycobacterium tuberculosis* infection, mitochondrial transplantation promotes the generation of protective effector and memory CD4^+^ T cells while reducing T-cell exhaustion and senescence-like phenotypes (88). However, mitochondria transfer can also suppress effector T-cell responses. Transfer of MSC-derived mitochondria to CD4^+^ T cells inhibits Th1 differentiation by downregulating T-bet (89), whereas transfer to CD8^+^ T cells suppresses T-bet and Eomes expression, reduces IFN-γ production, and impairs effector function (90). MSCs can also transfer mitochondria to allogeneic regulatory T cells in an HLA-dependent manner, thereby enhancing their immunosuppressive activity (91). In acute ischemic stroke, umbilical cord-derived MSCs regulate T-cell subset distribution and correct the Th17/Treg imbalance through mitochondria transfer, thereby alleviating peripheral immune dysregulation (92). In addition, MSC-mediated mitochondria transfer regulates B cell fate, indicating that its effects extend to broader adaptive immune networks (93). These findings demonstrate that mitochondria transfer does not uniformly activate or suppress adaptive immunity but instead reshapes metabolic fitness and effector responses according to the identity, activation state, and immunological function of the recipient lymphocyte.

In innate immunity, macrophages can serve as recipients and donors of mitochondria, as well as scavengers of extracellular mitochondrial material. Cardiac macrophage networks take up and eliminate abnormal mitochondrial material released by cardiomyocytes, forming an immune–parenchymal system that maintains myocardial mitochondrial homeostasis and organ function (43). Adipocyte-derived mitochondria are also transferred to macrophages to support white adipose tissue homeostasis, whereas this process is impaired in obesity (46). In the nervous system, macrophages transfer mitochondria to sensory neurons and promote the resolution of inflammatory pain (94). Platelet-derived extracellular vesicles containing mitochondria can also be internalized by neutrophils and alter their metabolic state and functional phenotype (84). Classic studies further demonstrated that MSCs transfer mitochondria to macrophages through TNTs, thereby enhancing macrophage bioenergetics and phagocytic activity and alleviating acute respiratory distress syndrome-related lung injury (28). MSCs can also deliver functional mitochondria to macrophages through extracellular vesicles, increasing oxidative phosphorylation and phagocytic capacity and improving lung injury (29). Moreover, MSCs can export mitochondria and associated cargo through extracellular vesicles. After uptake and reutilization by recipient macrophages, these mitochondria improve cellular bioenergetics and phagocytic function, thereby linking mitochondrial quality control in donor cells to innate immune remodeling in recipient cells (67). Collectively, these findings indicate that mitochondria-related transport contributes to innate immune surveillance, clearance of abnormal organelles, metabolic support, and the resolution of tissue inflammation.

Macrophage polarization represents an important interface through which mitochondria transfer links metabolic remodeling with immune regulation. In acute lung injury, MSC-derived extracellular vesicles transfer mitochondria or mitochondrial components to alveolar macrophages, restore mitochondrial membrane potential and oxidative phosphorylation, enhance phagocytic capacity, and promote an anti-inflammatory phenotype characterized by reduced IL-1β, TNF-α, and iNOS expression and increased IL-10 and Arg-1 expression (29, 95). Mitochondria transfer from MSCs to macrophages also suppresses renal inflammation in diabetic nephropathy through PGC-1α activation (66). Platelet-derived extracellular vesicles alleviate psoriasis-like inflammation by transferring mitochondria to macrophages (96). By contrast, mitochondria derived from inflammatory or damaged cells can promote pro-inflammatory macrophage reprogramming. Microvesicles released by LPS-primed macrophages transfer mitochondria to recipient macrophages, increase mitochondrial reactive oxygen species, activate the cGAS–STING–IFN-β pathway, promote an M1-like inflammatory phenotype, and reduce phagocytic capacity (48). Therefore, macrophage polarization is not determined solely by whether mitochondria transfer occurs but is strongly influenced by the quality and inflammatory state of the transferred mitochondria. Functional mitochondria can restore oxidative metabolism and promote inflammation resolution, whereas damaged or inflammation-conditioned mitochondria can act as metabolic and danger signals that reinforce pro-inflammatory macrophage activation.

Mitochondrial reactive oxygen species, cytosolic mtDNA sensing, inflammasome activation, and damage-associated signaling are major mechanisms through which mitochondrial transfer regulates inflammatory responses. After myocardial infarction, cardiac fibroblasts transfer damaged mitochondria to macrophages through mitochondria-enriched small extracellular vesicles, activating the NLRP3 pathway and promoting tissue inflammation and ventricular remodeling (47). During sepsis, microvesicles derived from pyroptotic macrophages promote neutrophil extracellular trap formation by delivering GSDMD-N-containing mitochondria to neutrophils, thereby inducing mitochondrial dysfunction and mitochondrial reactive oxygen species production (97). In osteoarthritis, elevated cholesterol promotes mitochondria transfer from subchondral osteocytes to chondrocytes, increases cytosolic mtDNA, and activates cGAS–STING signaling, thereby aggravating cartilage inflammation, synovitis, and joint degeneration (98). In inflammatory bowel disease, SM22α deficiency activates the RSAD2–YTHDF1 axis in colonic smooth muscle cells, inducing mitochondrial fragmentation and an inflammatory phenotype. These cells subsequently release extracellular vesicles containing mitochondrial components, which promote reactive oxygen species accumulation and ferroptosis in intestinal epithelial cells and aggravate colitis (99). Conversely, transfer of functional mitochondria can suppress inflammation in multiple disease contexts. Induced pluripotent stem cell–derived MSCs alleviate asthmatic inflammation through CX43-mediated mitochondria transfer (37). EPO-modified bone marrow MSCs enhance mitochondrial activation and transfer through HO-1 upregulation, thereby improving their anti-inflammatory effects (100). In neurological injury, hyperbaric oxygen preconditioning and MSC-loaded hydrogels reduce inflammatory responses by promoting mitochondria transfer (101, 102). In addition, mitochondria transfer from MSCs to osteoarthritic chondrocytes restores cellular function and alleviates inflammation and oxidative stress (103). Mitochondria-loaded erythrocytes attenuate inflammatory bone loss through mitochondrial delivery (104). These findings indicate that the inflammatory effects of mitochondria transfer vary according to mitochondrial quality, promoting tissue repair when functional mitochondria are transferred but aggravating injury when damaged mitochondria or mtDNA activate innate immune signaling.

The diverse roles of mitochondria transfer are particularly evident in the tumor microenvironment, where it regulates tumor metabolic adaptation, antitumor immunity, and immune evasion. Studies have shown that TNTs can form between tumor cells and immune cells and mediate mitochondrial trafficking between these cell populations, thereby enhancing the metabolic capacity of tumor cells while impairing immune cell function (105). Mitochondria transfer from immune cells to tumor cells can also promote lymph node metastasis and induce a cGAS–STING-associated type I interferon program that supports metastatic adaptation (106). Mitochondria can also be transferred in the opposite direction, from tumor cells to immune cells. Tumor cells transfer mitochondria carrying mtDNA mutations and mitophagy-inhibitory molecules to tumor-infiltrating T cells. These transferred mitochondria evade mitophagic clearance and progressively replace endogenous T-cell mitochondria, resulting in metabolic dysfunction, senescence-like changes, impaired effector function, and defective memory formation, ultimately promoting tumor immune evasion and resistance to immune checkpoint therapy (45). In addition, mtDNA released by senescent tumor cells can be taken up by polymorphonuclear myeloid–derived suppressor cells and contribute to the formation of an immunosuppressive tumor microenvironment through the cGAS–STING pathway (107). However, STING activation does not invariably promote tumor progression. Osteocyte-derived mitochondria transferred to metastatic tumor cells can activate STING-dependent antitumor immunity and suppress tumor development (108). Therefore, the effects of mitochondria transfer in cancer depend on the direction of transfer, mitochondrial quality, recipient cell identity, and the local immune microenvironment.

In summary, the transfer of functional mitochondria generally helps restore oxidative phosphorylation, maintain the metabolic fitness of immune cells, and promote inflammation resolution. In contrast, the transfer of damaged mitochondria, mutated mtDNA, or inflammation-related mitochondrial components may activate cGAS–STING, NLRP3, mitochondrial reactive oxygen species, and other danger-signaling pathways, thereby promoting immunosuppression or inflammatory tissue injury. Future studies should further distinguish the transfer of intact functional mitochondria from the transport of mitochondrial fragments, mtDNA, and damage-associated components, clarify the direction and cell specificity of mitochondria transfer *in vivo*, and determine how mitochondrial quality, the immune state of recipient cells, and the local microenvironment collectively shape its immunological and inflammatory effects.

#### 4.2.4 Mitochondrial transplantation

Bibliometric analysis identifies mitochondrial transplantation as one of the clearest translational directions in mitochondria transfer research. In the co-cited reference clustering and keyword analyses, mitochondrial transplantation was closely associated with mitochondrial transfer, artificial mitochondria transfer, intercellular mitochondrial transport, therapy, regenerative medicine, extracellular mitochondria, and neuroprotection, indicating its central position in the translational knowledge structure of this field. Unlike naturally occurring intercellular mitochondria transfer, artificial mitochondrial transplantation places greater emphasis on key steps, including the *in-vitro* isolation of donor mitochondria, maintenance of mitochondrial activity, delivery precision and efficiency, uptake by recipient cells, and functional integration after transplantation (17). With advances in related technologies, mitochondrial transplantation has gradually evolved from simple exogenous mitochondrial delivery into a multilayered biotechnological therapeutic strategy integrating natural vesicles, extracellular vesicles, biomaterials, nanocarriers, and engineered targeting systems.

Mitochondrial surface modification, natural vesicle-based delivery, and related strategies are among the key approaches for enhancing mitochondrial stability and cellular uptake efficiency. Recently, cell-free mitochondrial therapy has emerged as a new concept in which isolated mitochondria are regarded as “active biologics” or organelle-based therapeutics that can be directly delivered to diseased tissues to restore bioenergetic and redox homeostasis (109). This concept has been supported by recent studies using MSC-derived mitochondria in osteoarthritis and plasma-derived mitochondria in postoperative paraspinal muscle atrophy, which further demonstrate the feasibility, safety, and accessibility of cell-free mitochondrial transplantation (110, 111). To improve mitochondrial delivery efficiency, artificial lipid membrane coating has been shown to enhance mitochondrial surface stability and cellular uptake capacity, suggesting its potential to improve the therapeutic efficacy of allogeneic mitochondrial transplantation (112). Cell-penetrating peptide Pep-1–mediated mitochondrial delivery has also demonstrated high delivery efficiency in cybrid models related to inherited mitochondrial diseases, improving mitochondrial respiratory function, oxidative stress tolerance, and cell survival (113, 114). In addition to artificial modification, natural vesicles can serve as carriers for mitochondrial delivery. For example, synaptosomes contain synaptic structures and functional mitochondria, can be taken up by neuron-like cells, and can restore mitochondrial activity in models of mitochondrial injury, suggesting their potential as natural carriers for mitochondrial transplantation in the nervous system (115). Similarly, intramyocardial injection of mitochondria-rich extracellular vesicles released by autologous stem cell–derived cardiomyocytes can transfer mitochondria into hypoxia-injured cardiomyocytes, improve intracellular energy metabolism, and enhance cardiac function after myocardial infarction (72).

In addition, engineered mitochondrial delivery systems represent a useful strategy to improve the stability, cellular uptake, tissue targeting, and retention of transplanted mitochondria. Early physical and cell-engineering approaches, including MitoCeption, photothermal nanoblade-mediated delivery, MitoPunch, and magnetomitotransfer, enable quantitative mitochondrial delivery, transient membrane permeabilization, pressure-driven delivery, and magnetically guided delivery, respectively, thereby improving transfer efficiency and controllability for *in-vitro* or *ex-vivo* mitochondrial engineering (116–119). For *in-vivo* applications, nanocarrier- and biomaterial-based systems have been developed to improve mitochondrial protection, targeting, and therapeutic persistence. Fusogenic liposome-encapsulated mitochondria have been investigated for osteoarthritis treatment, with membrane fusion properties used to improve mitochondrial delivery to chondrocytes (120). Nitric oxide-releasing nanomotor-modified mitochondria have been developed for oral mitochondrial transplantation, enabling intestinal release, systemic delivery, accumulation in injured cardiac tissue, and metabolic improvement in ischemic heart disease (121). Intravenous administration of ischemia-targeting peptide–TPP–mitochondria complexes represents a strategy for targeted delivery to ischemic tissues and can be used to alleviate myocardial ischemia–reperfusion injury (122). Another study constructed engineered mitochondrial ROS-scavenging nanocomplexes by fusing mitochondria with liposomes, thereby enhancing pulmonary biodistribution and reducing inflammatory responses (123). Local biomaterial depots, particularly hydrogel-based systems, further improve mitochondrial retention and sustained release at injured sites. Alginate-based hydrogels loaded with MSC-derived mitochondria can promote angiogenesis in a rat model of acute myocardial infarction (124). Methylcellulose/hyaluronic acid thermogels can release approximately 70% of loaded mitochondria within 20 min at 37°C, and the released mitochondria retain strong respiratory capacity, supporting their use for local spinal cord transplantation (125). Degalactosylated xyloglucan hydrogels exhibit good injectability and mitochondrial embedding capacity, which may improve the stability of mitochondrial transplantation (126). Liquid–liquid phase separation hydrogels can concentrate and protect freshly isolated mitochondria through a gelatin–PEG system, maintain mitochondrial membrane potential and ATP production, promote cardiomyocyte uptake in a myocardial ischemia–reperfusion model, and reduce tissue injury (127). More recently, precision-engineered systems have further advanced mitochondrial transplantation toward cell type–specific and functionally stable organelle therapy. The MitoCatch system enables programmable delivery of donor mitochondria to specific cell types through protein binders. This system promotes donor mitochondrial entry into recipient cells and allows subsequent cytoplasmic trafficking, fusion, and fission (128). In addition, erythrocyte membrane-derived mitochondrial capsules markedly improve delivery efficiency and functional stability, promote donor mitochondrial integration into the endogenous mitochondrial network, and rescue mitochondrial dysfunction in mitochondrial disease and Parkinson’s disease models (129). Beyond delivery efficiency, recent studies have highlighted that the functional outcome of mitochondrial transplantation depends not only on mitochondrial uptake, intracellular trafficking, and functional integration, but also on mitochondrial quality, metabolic compatibility, and donor–recipient mitochondrial competition (130). Mitophagy acts as a context-dependent regulator of post-transplantation mitochondrial fate: it promotes endothelial engraftment and revascularization through PINK1–Parkin signaling in one setting (42), but excessive mitophagy is reduced after mitochondrial transplantation in cerebellar neurodegeneration, contributing to improved mitochondrial function and delayed neuronal apoptosis (131). In addition to intracellular mitochondrial fate, mitochondrial transplantation may also reshape the inflammatory and immunometabolic microenvironment, as mitochondrial delivery systems based on engineered macrophages or nanoparticles can modulate immune responses in ischemic and pain-related disease models (132, 133). Consistently, mitochondrial transplantation has also been shown to ameliorate rheumatoid arthritis by regulating abnormal cGAS–STING1 signaling activation, autophagosome accumulation, and necroptosis, further supporting its role in immune–inflammatory modulation (134).

According to an updated review of ClinicalTrials.gov records and available clinical reports, mitochondrial transplantation-based therapies remain at an early stage of clinical translation and are still mainly represented by small-sample, early phase exploratory studies. NCT02851758 and NCT04998357 evaluated the safety and feasibility of isolating mitochondria from autologous skeletal muscle tissue and delivering them to ischemic myocardium or the vascular territory affected by acute cerebral ischemia, respectively. Although pilot clinical data have suggested acceptable feasibility and no major procedure-related safety signals (135), their value as clinical evidence should be interpreted cautiously given the early phase, open-label, or small-sample nature of these studies. NCT04976140 evaluated PN-101, an allogeneic mitochondrial product derived from human umbilical cord mesenchymal stem cells, in patients with refractory polymyositis or dermatomyositis. This completed phase 1/2a study reported no severe adverse drug reactions and preliminary improvement signals, but the clinical outcomes remain inconclusive because of the limited sample size (136). Its subsequent trial, NCT07122648, is registered as a multicenter, randomized, double-blind, placebo-controlled phase II study designed to further evaluate the efficacy and safety of PN-101. NCT05669144 explored the co-transplantation of MSC-derived exosomes and autologous mitochondria in patients undergoing coronary artery bypass grafting, but no mature clinical outcomes have yet been reported. NCT06017869 is an active phase II study investigating MNV-201, an autologous CD34^+^ cell product augmented with allogeneic placenta-derived mitochondria, for Pearson syndrome. Overall, current clinical evidence remains largely limited to safety, feasibility, and preliminary efficacy signals, and the therapeutic value of mitochondrial transplantation requires further validation through larger multicenter randomized controlled trials and long-term follow-up.

### 4.3 Limitations

Although this study provides a bibliometric and visualization-based overview of mitochondria transfer-related research over the past two decades, several limitations should be acknowledged. First, although the combined use of WoSCC and Scopus improved literature coverage, differences in database scope, indexing formats, and update frequency may have introduced selection bias, and relevant studies indexed elsewhere may have been missed. Second, only English-language publications were included, which may have underestimated high-quality studies published in other languages. Third, because the search was conducted on April 8, 2026, publication data for 2026 were incomplete and should be interpreted cautiously. Citation-based indicators are also influenced by citation lag, whereby recently published studies may not yet have accumulated sufficient citations, whereas earlier publications may have citation advantages. In addition, despite manual screening by two independent researchers, some relevant studies may have been omitted because of inconsistent terminology or atypical descriptions, and a degree of subjectivity in study selection could not be fully avoided. Finally, bibliometric indicators, including publication counts, citation frequency, centrality, and keyword frequency, mainly reflect academic visibility and network position rather than mechanistic importance or clinical significance.

## 5 Conclusion

In conclusion, mitochondria transfer has emerged as a rapidly expanding interdisciplinary field linking mitochondrial biology, intercellular communication, immune–inflammatory regulation, disease mechanisms, and translational therapy. Bibliometric and visualization analyses indicate that this field has evolved from early studies of transfer phenomena and stem cell–mediated rescue toward broader cellular mechanisms, including mitochondrial quality control, metabolic remodeling, and functional integration of transferred mitochondria. The growing focus on macrophages, T cells, B cells, immune evasion, macrophage polarization, inflammatory responses, tumor microenvironment remodeling, and cGAS–STING-related mechanisms further highlights immune–inflammatory regulation as an important frontier, suggesting that mitochondria transfer functions as a context-dependent form of immunometabolic communication.

Future studies should further clarify the molecular determinants of different mitochondria transfer routes and determine how donor cells, recipient cells, the functional status of mitochondria, and disease microenvironments jointly shape the outcomes of mitochondria transfer. With respect to mitochondrial quality control, particular attention should be given to how mitochondrial biogenesis, fission and fusion, mitophagy, and post-transfer functional integration regulate mitochondrial selection, delivery, utilization, and clearance. In particular, it is necessary to distinguish the protective transfer of functional mitochondria from the deleterious transfer of damaged mitochondria, mitochondrial fragments, or mitochondria carrying mutant mtDNA. With respect to metabolic homeostasis, future studies should further determine how mitochondria transfer reshapes oxidative phosphorylation, glycolysis, redox balance, lipid metabolism, and metabolic flexibility. It is also necessary to determine whether these effects represent transient bioenergetic compensation or induce sustained metabolic reprogramming. On this basis, it is also necessary to clarify how different metabolic changes affect immune cell activation, polarization, exhaustion, inflammatory signaling, and tumor immune evasion, thereby revealing the causal relationships among mitochondrial quality control, metabolic remodeling, and immune regulation.

In addition, standardized methods for dynamic tracking, quantitative evaluation, and functional validation are needed. With respect to mitochondrial transplantation, future studies should optimize mitochondrial isolation, preservation, targeted delivery, and functional integration processes and establish unified criteria for evaluating mitochondrial quality and therapeutic potency. Engineered vesicles, biomaterials, nanocarriers, and cell-specific targeting systems may improve mitochondrial stability, delivery efficiency, and targeting precision, but their biocompatibility and long-term safety require further validation. Future studies should also conduct large-scale, multicenter, randomized controlled trials with long-term follow-up to further determine the safety, clinical efficacy, and applicable disease spectrum of mitochondrial transplantation.

## Data Availability

The original contributions presented in the study are included in the article/**Supplementary Material**. Further inquiries can be directed to the corresponding authors.
