# Supplementary material for: Mitochondria transfer research from cellular mechanisms to immune–inflammatory and translational frontiers: a bibliometric analysis from 2006 to 2026
Source: Front Immunol. 2026 Aug 19;17:1898280. doi: 10.3389/fimmu.2026.1898280 (PMC13533918; doi:10.3389/fimmu.2026.1898280)
Supplement: Supplementary file 1 [file Table1.docx]

Both Web of Science (WoS) and Scopus search queries employed title, abstract, and keyword retrieval.

Search terms for mitochondria transfer were determined based on Reference (Recommendations for mitochondria transfer and transplantation nomenclature and characterization https://doi.org/10.1038/s42255-024-01200-x).

During preliminary retrieval, mitochondrial transfer RNA was identified as a source of retrieval noise; accordingly, terms related to mitochondrial transfer RNA were excluded. Finally, we manually reviewed the literature that had been excluded based on the mitochondrial transfer RNA‑related search terms.

WoS search query:

( TI=("mitochondria transfer" OR "mitochondrial transfer" OR "intercellular mitochondria transfer" OR "intercellular mitochondrial transfer" OR "horizontal mitochondria transfer" OR "horizontal mitochondrial transfer") OR AB=("mitochondria transfer" OR "mitochondrial transfer" OR "intercellular mitochondria transfer" OR "intercellular mitochondrial transfer" OR "horizontal mitochondria transfer" OR "horizontal mitochondrial transfer") OR AK=("mitochondria transfer" OR "mitochondrial transfer" OR "intercellular mitochondria transfer" OR "intercellular mitochondrial transfer" OR "horizontal mitochondria transfer" OR "horizontal mitochondrial transfer") ) NOT ( TI=("mitochondrial transfer RNA" OR "mitochondrial tRNA" OR "mitochondrial transfer RNAs" OR "mitochondrial tRNAs") OR AB=("mitochondrial transfer RNA" OR "mitochondrial tRNA" OR "mitochondrial transfer RNAs" OR "mitochondrial tRNAs") OR AK=("mitochondrial transfer RNA" OR "mitochondrial tRNA" OR "mitochondrial transfer RNAs" OR "mitochondrial tRNAs") )

View the Web of Science search query that has excluded mitochondrial transfer RNA:

( TI=("mitochondria transfer" OR "mitochondrial transfer" OR "intercellular mitochondria transfer" OR "intercellular mitochondrial transfer" OR "horizontal mitochondria transfer" OR "horizontal mitochondrial transfer") OR AB=("mitochondria transfer" OR "mitochondrial transfer" OR "intercellular mitochondria transfer" OR "intercellular mitochondrial transfer" OR "horizontal mitochondria transfer" OR "horizontal mitochondrial transfer") OR AK=("mitochondria transfer" OR "mitochondrial transfer" OR "intercellular mitochondria transfer" OR "intercellular mitochondrial transfer" OR "horizontal mitochondria transfer" OR "horizontal mitochondrial transfer") ) AND ( TI=("mitochondrial transfer RNA" OR "mitochondrial tRNA" OR "mitochondrial transfer RNAs" OR "mitochondrial tRNAs") OR AB=("mitochondrial transfer RNA" OR "mitochondrial tRNA" OR "mitochondrial transfer RNAs" OR "mitochondrial tRNAs") OR AK=("mitochondrial transfer RNA" OR "mitochondrial tRNA" OR "mitochondrial transfer RNAs" OR "mitochondrial tRNAs") )

Scopus search query:

TITLE-ABS-KEY(

("mitochondria transfer" OR "mitochondrial transfer" OR "intercellular mitochondria transfer" OR "intercellular mitochondrial transfer" OR "horizontal mitochondria transfer" OR "horizontal mitochondrial transfer")

AND NOT

("mitochondrial transfer RNA" OR "mitochondrial tRNA" OR "mitochondrial transfer RNAs" OR "mitochondrial tRNAs")

)

View the Scopus search query that has excluded mitochondrial transfer RNA:

TITLE-ABS-KEY(

("mitochondria transfer" OR "mitochondrial transfer" OR

"intercellular mitochondria transfer" OR "intercellular mitochondrial transfer" OR

"horizontal mitochondria transfer" OR "horizontal mitochondrial transfer")

AND

("mitochondrial transfer RNA" OR "mitochondrial tRNA" OR

"mitochondrial transfer RNAs" OR "mitochondrial tRNAs")

)
